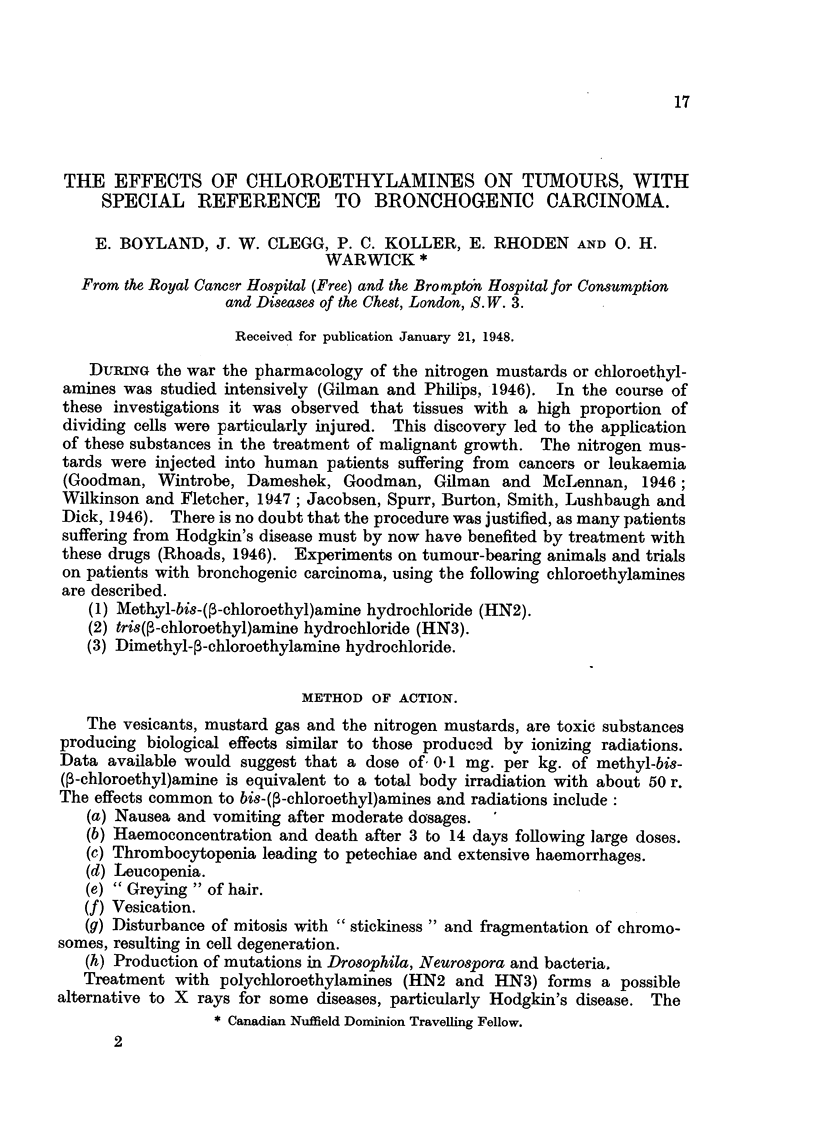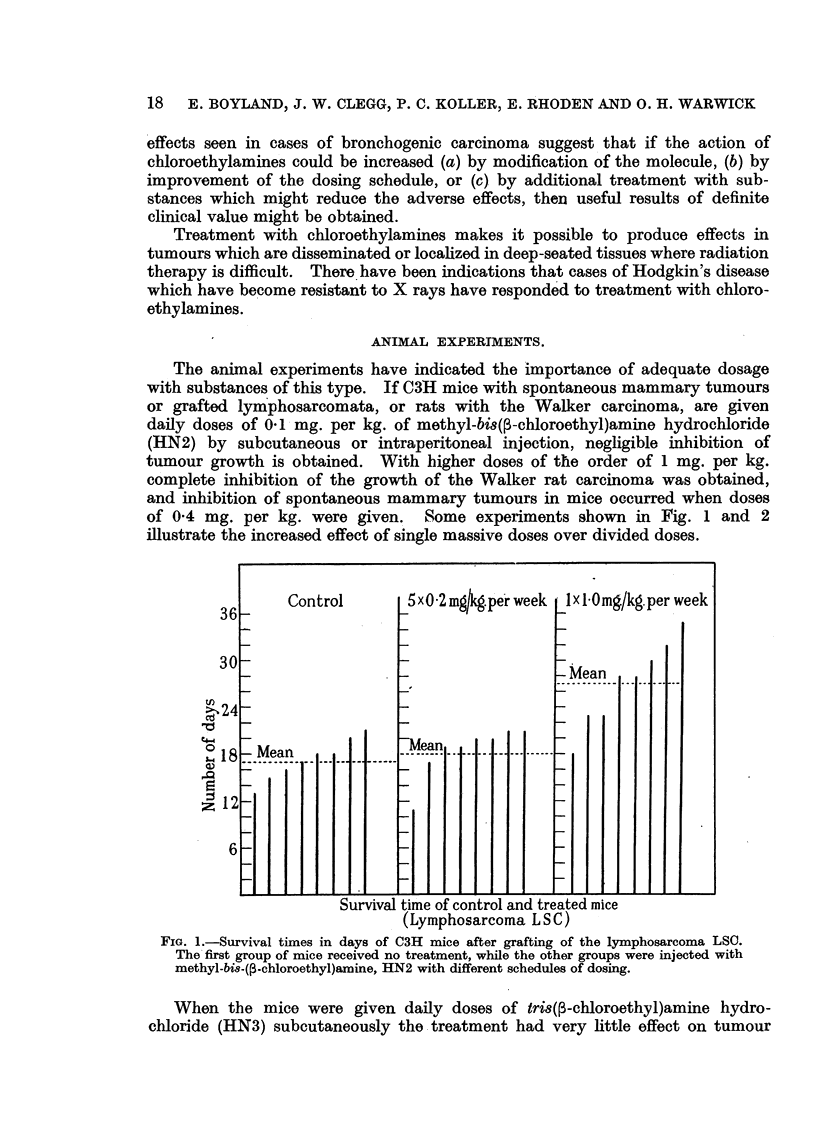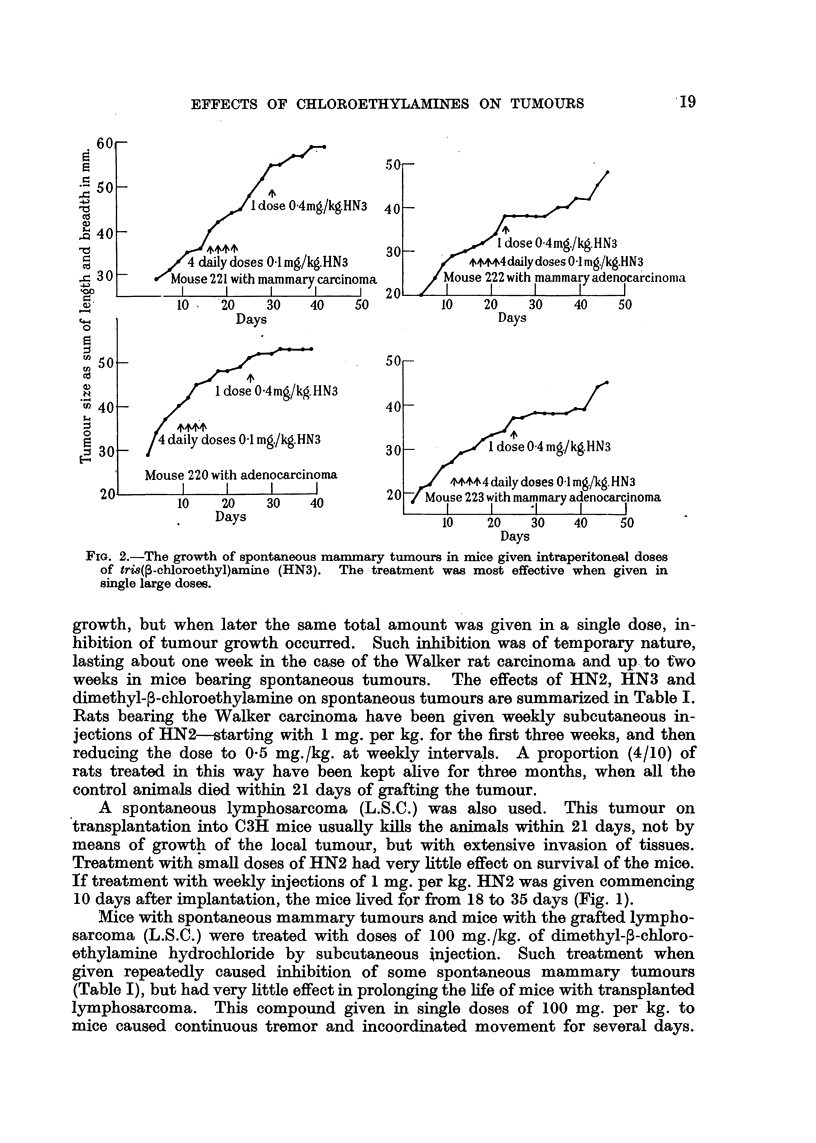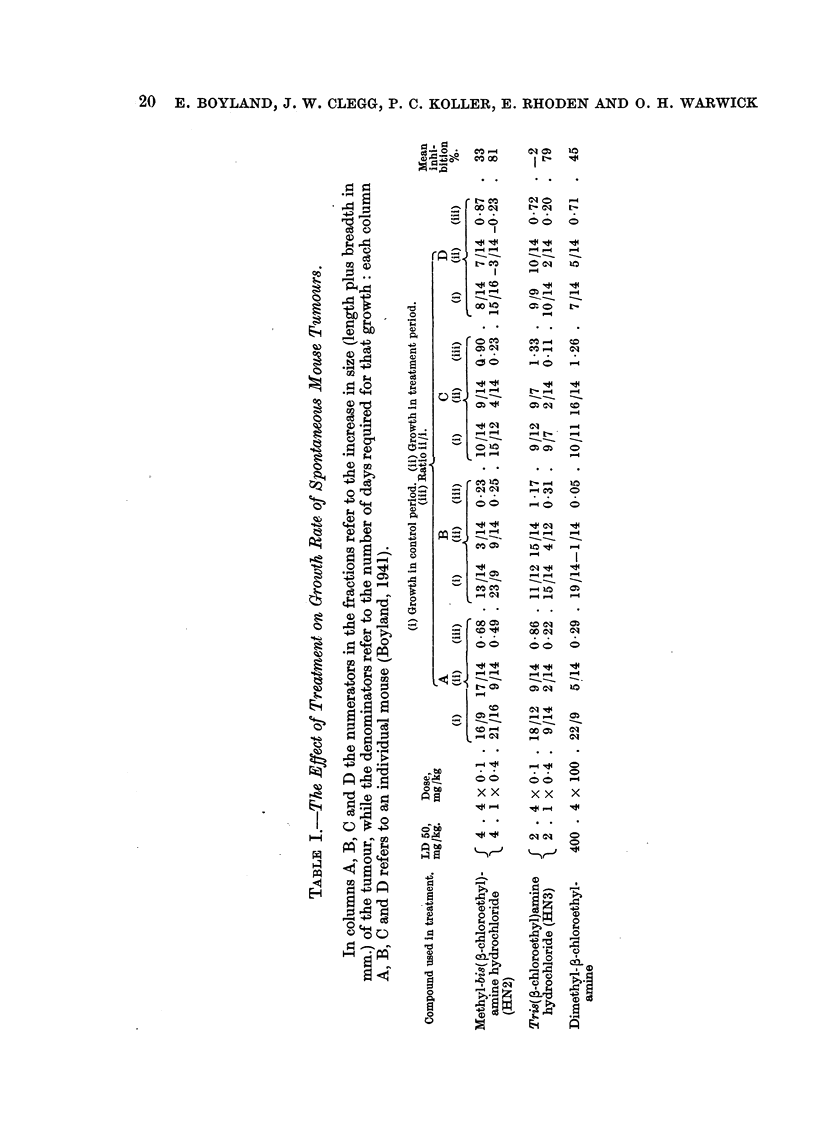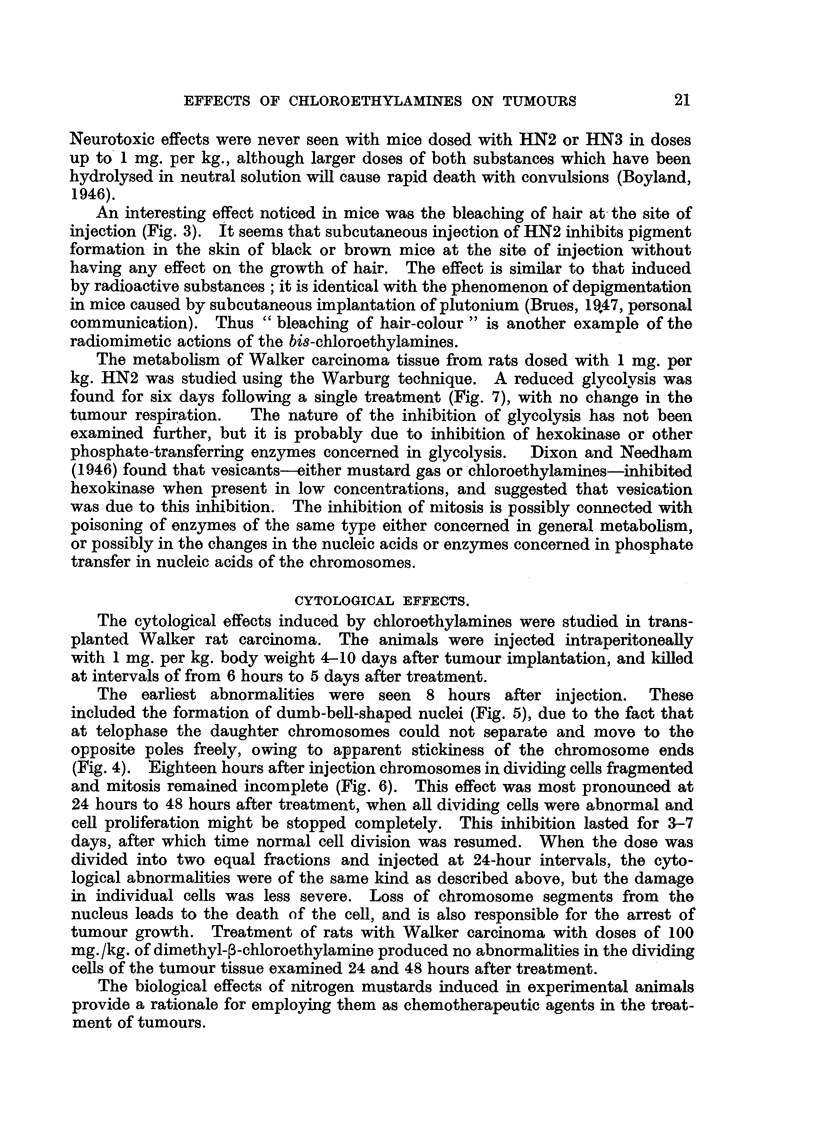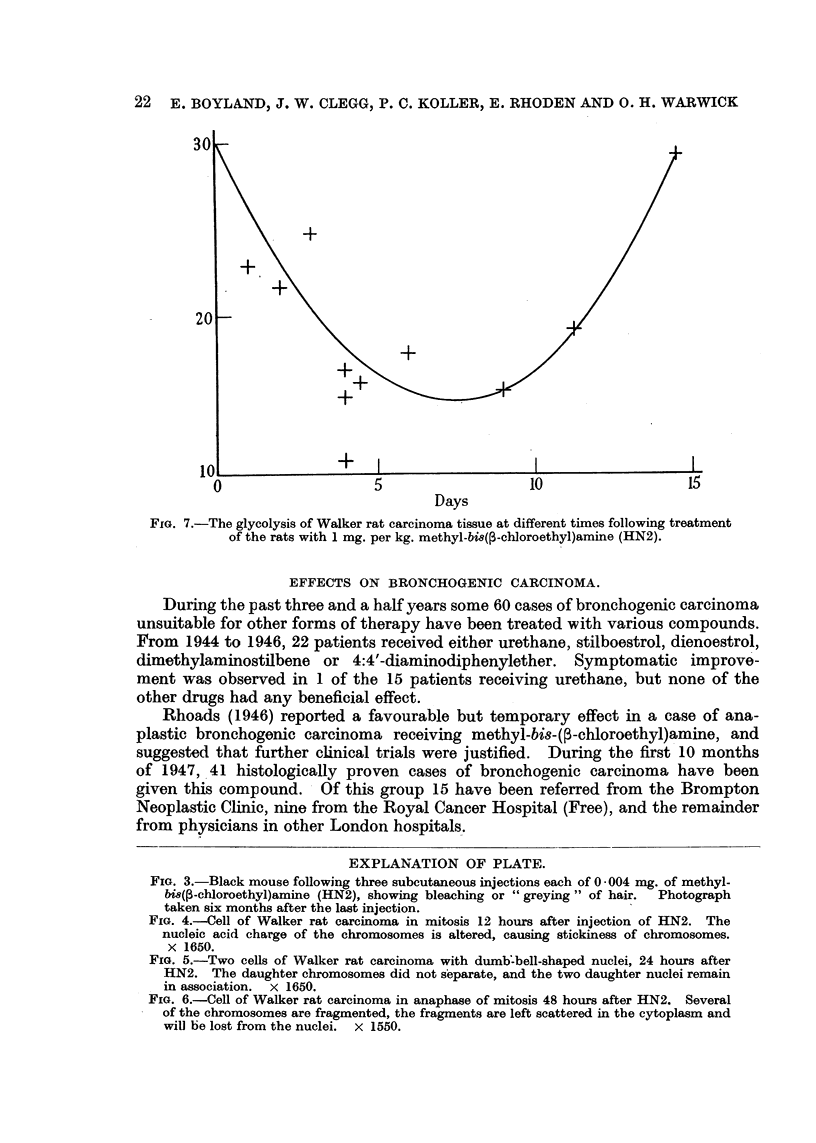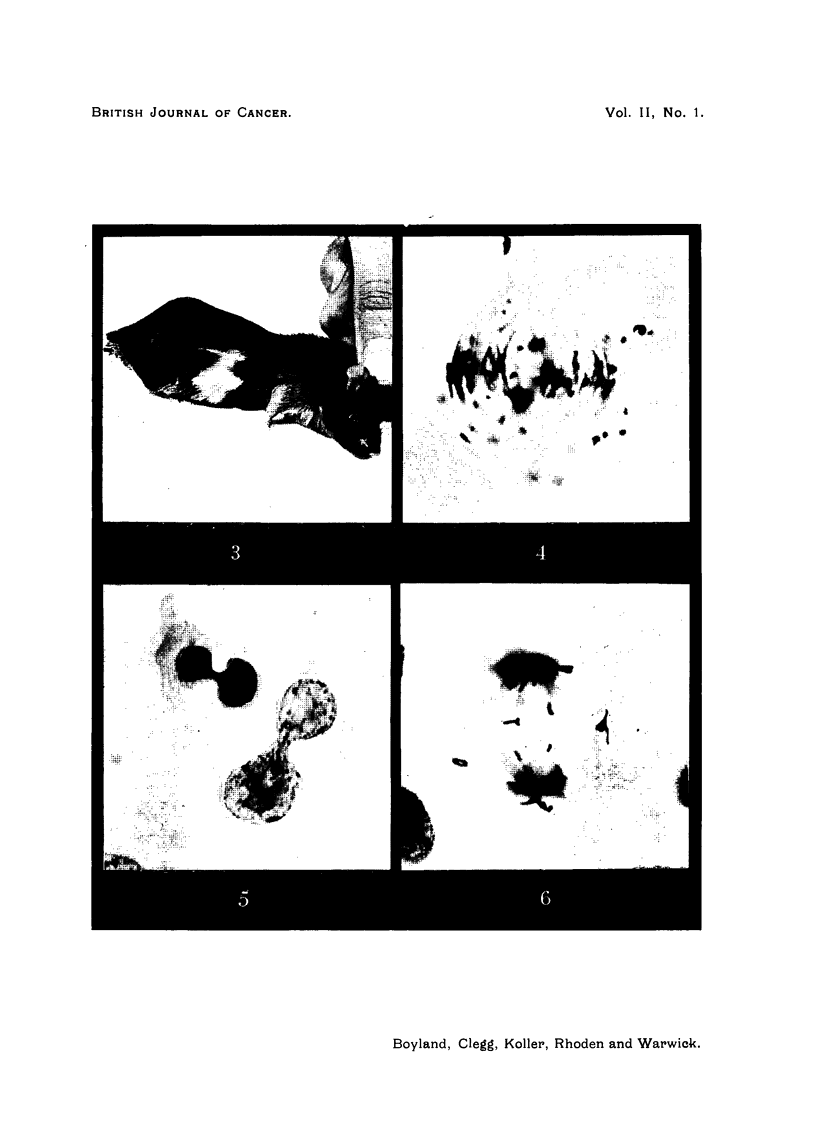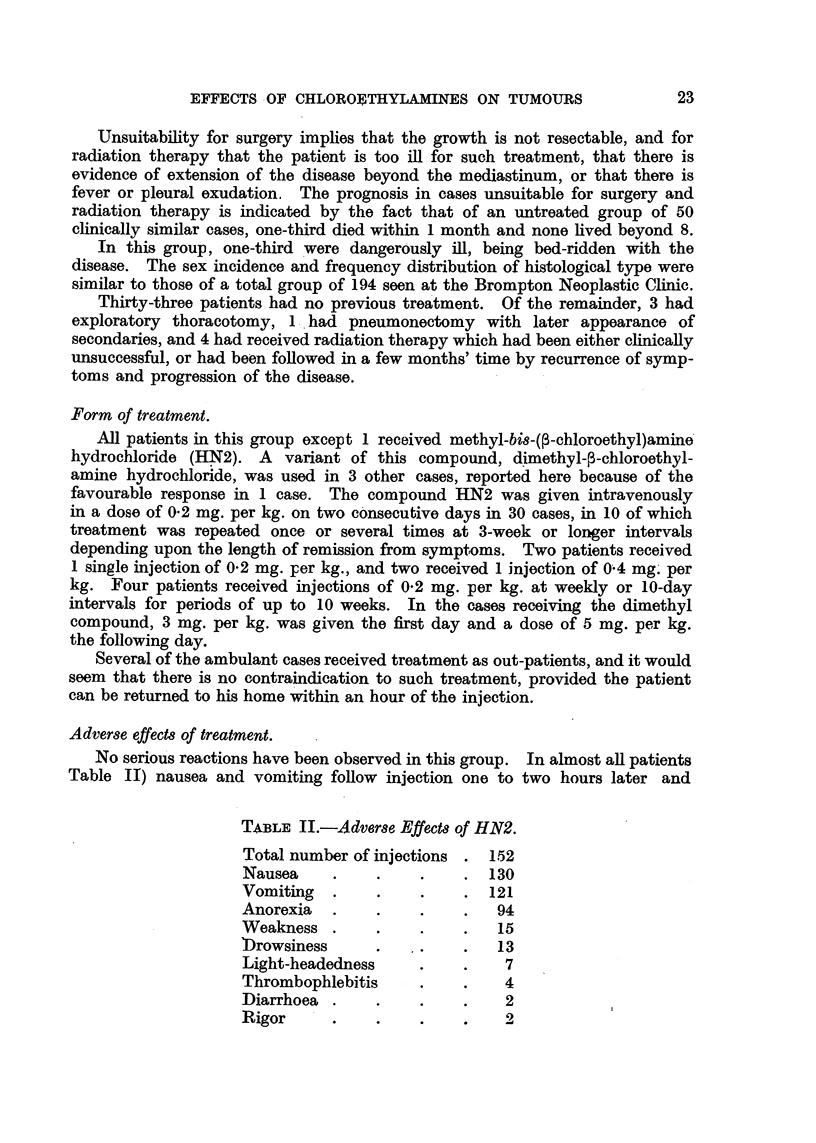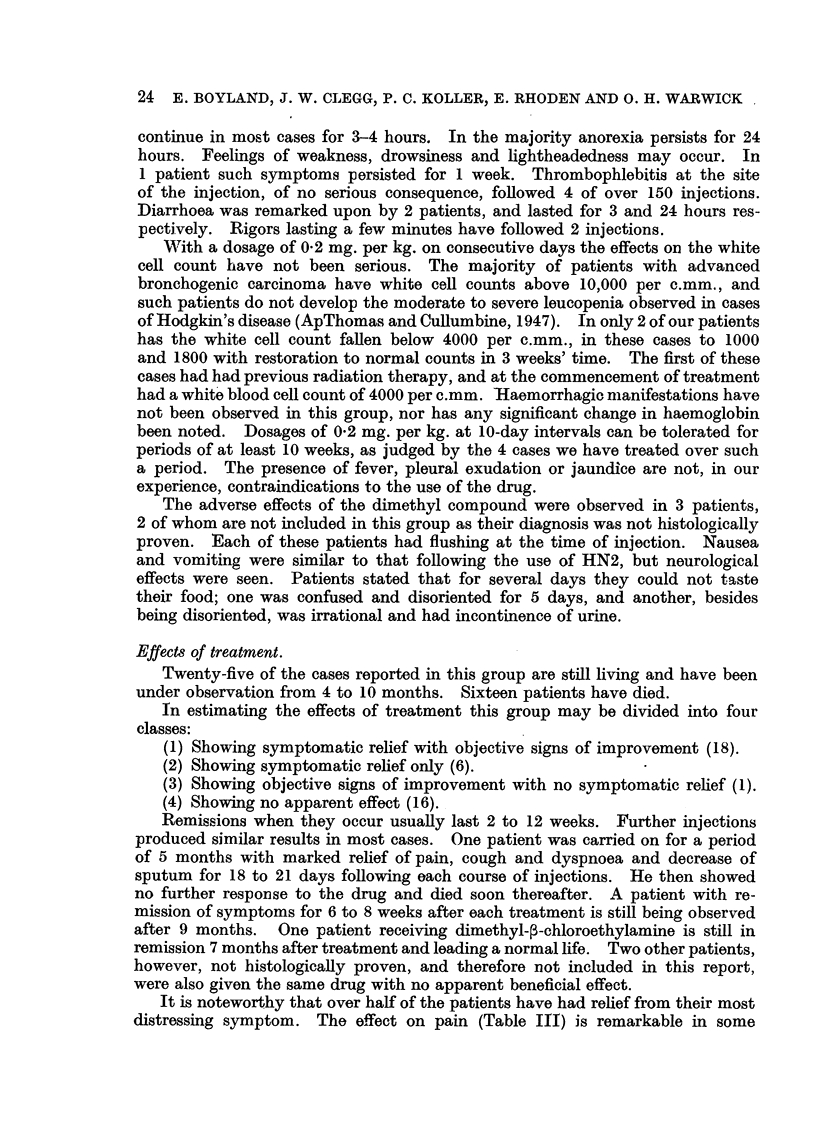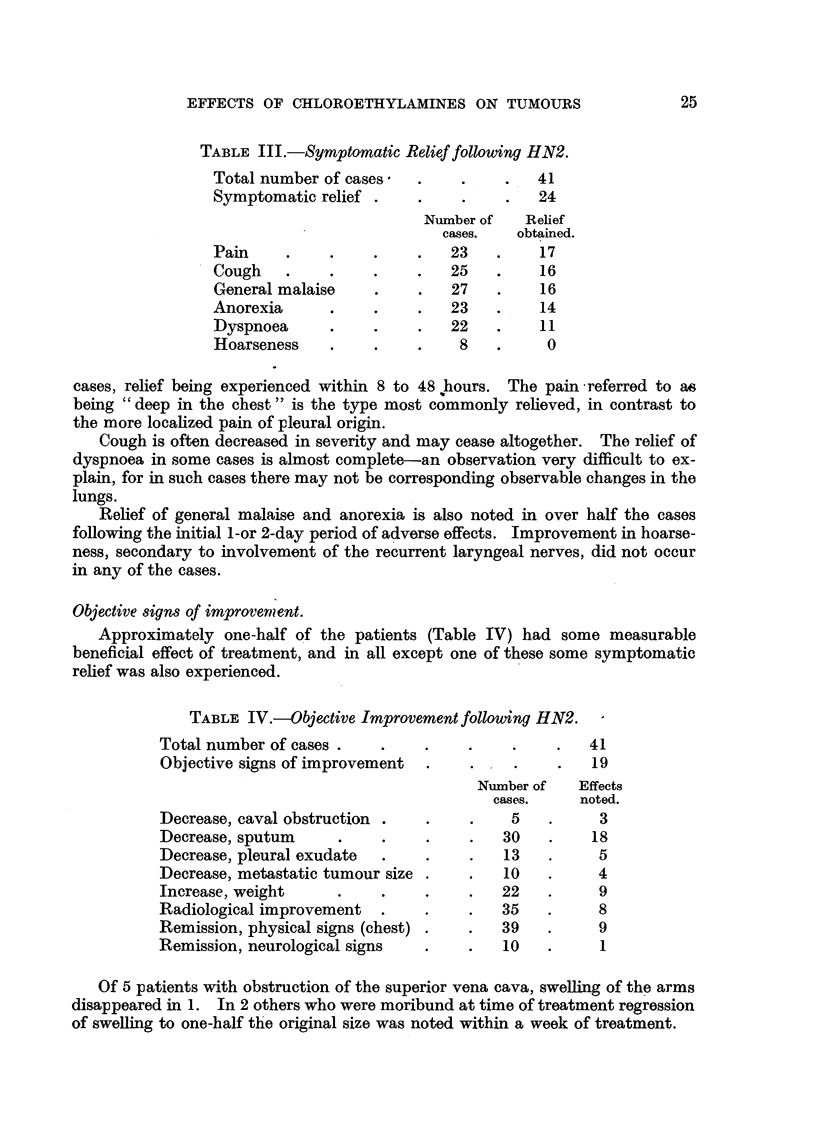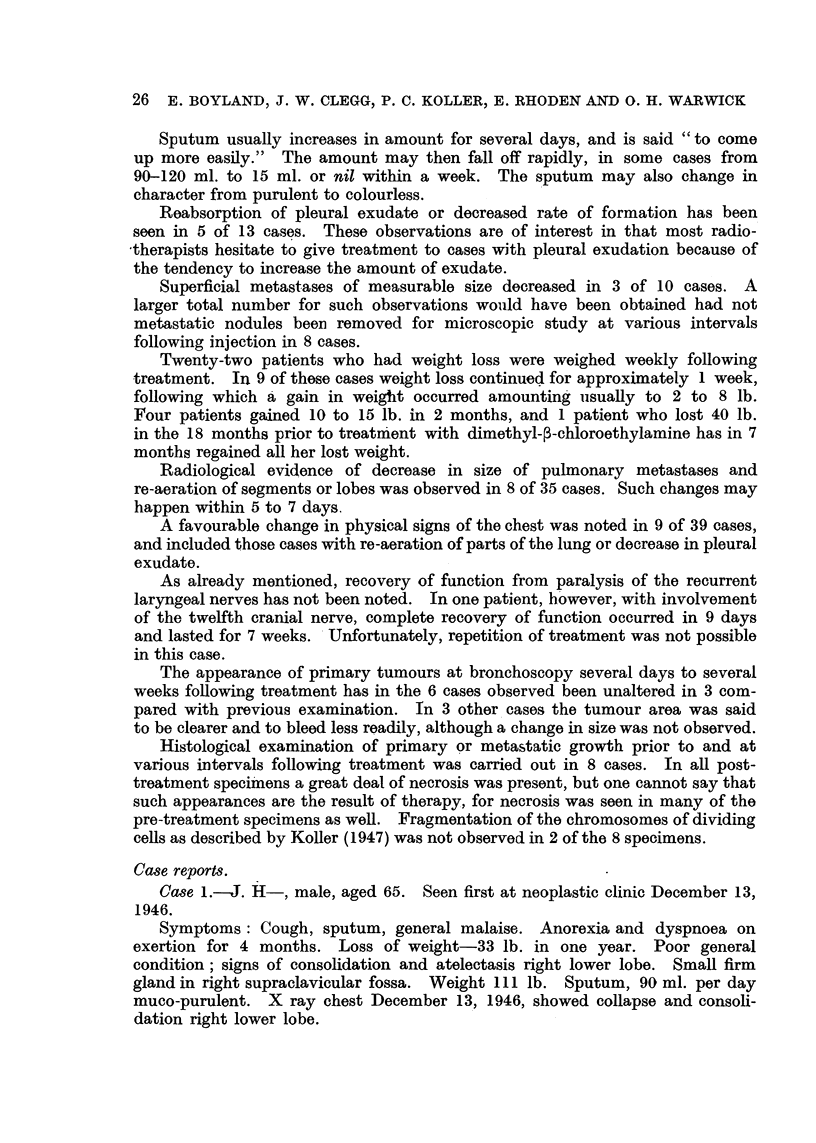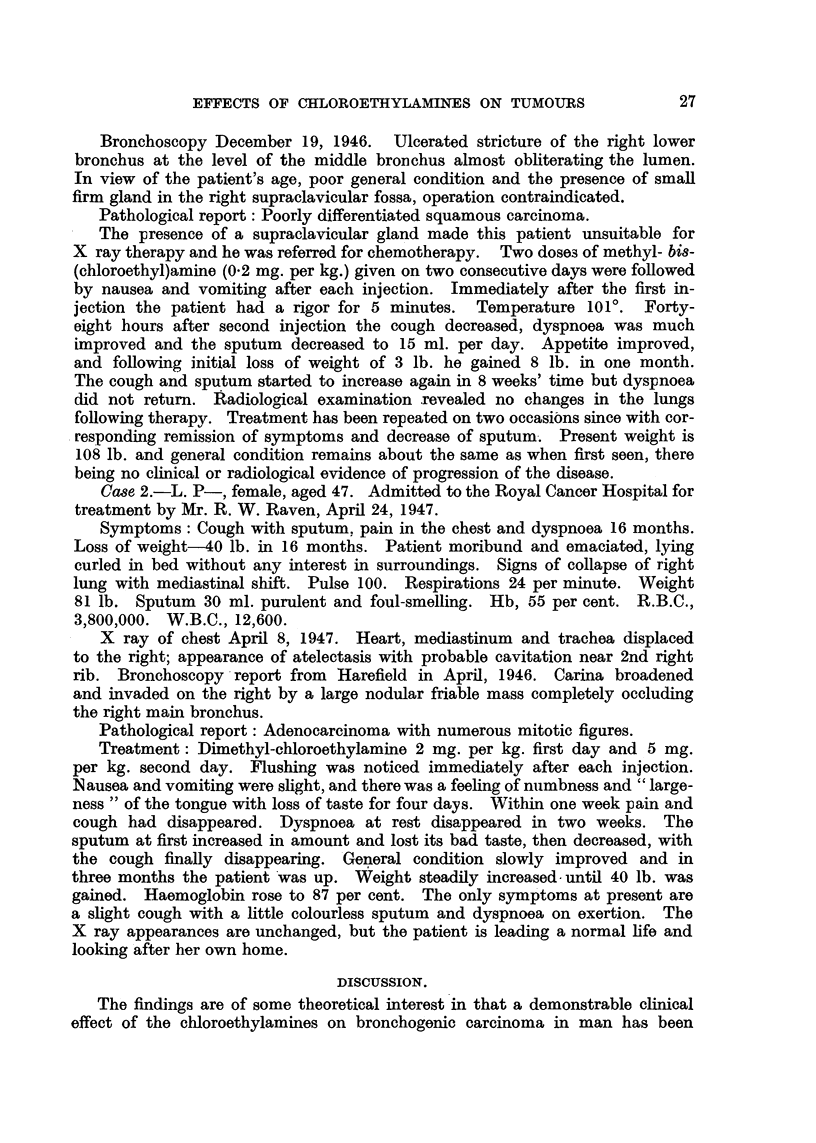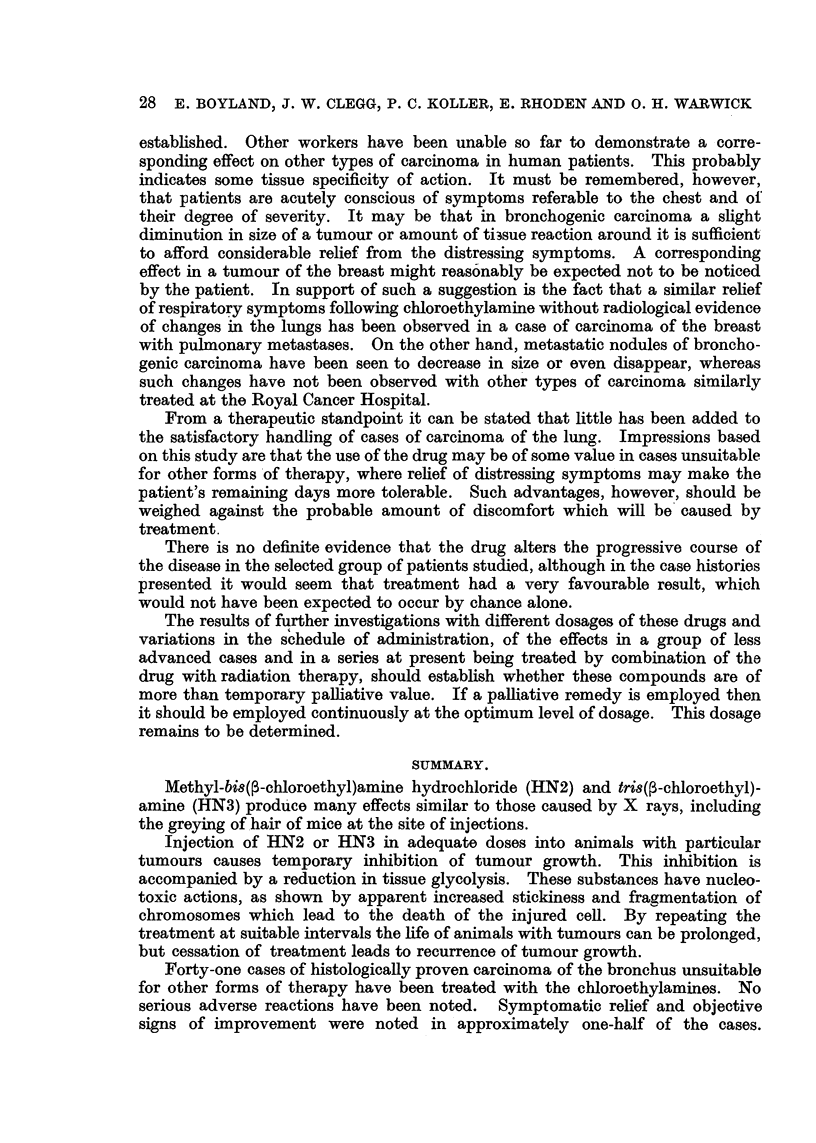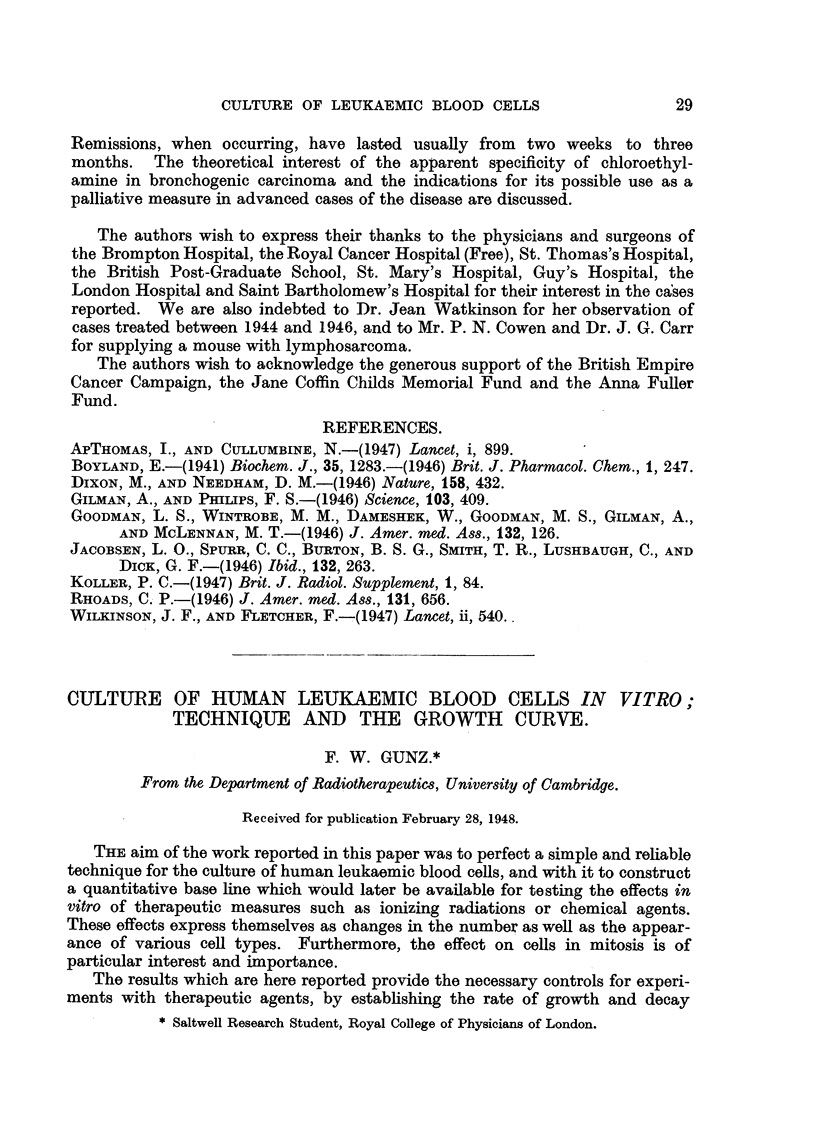# The Effects of Chloroethylamines on Tumours, with Special Reference to Bronchogenic Carcinoma

**DOI:** 10.1038/bjc.1948.4

**Published:** 1948-03

**Authors:** E. Boyland, J. W. Clegg, P. C. Koller, E. Rhoden, O. H. Warwick

## Abstract

**Images:**


### THE EFFECTS OF CHLOROETHYLAMINES ON TUMOURS, WITH SPECIAL REFERENCE TO BRONCHOGENIC CARCINOMA.

E. BOYLAND, J. W. CLEGG, P. C. KOLLER, E. RHODEN AND O. H. WARWICK *

*From the Royal Cancer Hospital (Free) and the Brompton Hospital for Consumption and Diseases of the Chest, London, S.W. 3.*

Received for publication January 21, 1948.

DURING the war the pharmacology of the nitrogen mustards or chloroethylamines was studied intensively (Gilman and Philips, 1946). In the course of these investigations it was observed that tissues with a high proportion of dividing cells were particularly injured. This discovery led to the application of these substances in the treatment of malignant growth. The nitrogen mustards were injected into human patients suffering from cancers or leukaemia (Goodman, Wintrobe, Dameshek, Goodman, Gilman and McLennan, 1946; Wilkinson and Fletcher, 1947; Jacobsen, Spurr, Burton, Smith, Lushbaugh and Dick, 1946). There is no doubt that the procedure was justified, as many patients suffering from Hodgkin's disease must by now have benefited by treatment with these drugs (Rhoads, 1946). Experiments on tumour-bearing animals and trials on patients with bronchogenic carcinoma, using the following chloroethylamines are described.

(1) Methyl-*bis*-(β-chloroethyl)amine hydrochloride (HN2).

(2) *tris*(β-chloroethyl)amine hydrochloride (HN3).

(3) Dimethyl-β-chloroethylamine hydrochloride.

### METHOD OF ACTION.

The vesicants, mustard gas and the nitrogen mustards, are toxic substances producing biological effects similar to those produced by ionizing radiations. Data available would suggest that a dose of 0·1 mg. per kg. of methyl-*bis*-(β-chloroethyl)amine is equivalent to a total body irradiation with about 50 r. The effects common to *bis*-(β-chloroethyl)amines and radiations include:

(*a*) Nausea and vomiting after moderate dosages.
(*b*) Haemoconcentration and death after 3 to 14 days following large doses.
(*c*) Thrombocytopenia leading to petechiae and extensive haemorrhages.
(*d*) Leucopenia.
(*e*) " Greying " of hair.
(*f*) Vesication.
(*g*) Disturbance of mitosis with " stickiness " and fragmentation of chromosomes, resulting in cell degeneration.
(*h*) Production of mutations in *Drosophila*, *Neurospora* and bacteria.

Treatment with polychloroethylamines (HN2 and HN3) forms a possible alternative to X rays for some diseases, particularly Hodgkin's disease. The

* Canadian Nuffield Dominion Travelling Fellow.

effects seen in cases of bronchogenic carcinoma suggest that if the action of chloroethylamines could be increased (*a*) by modification of the molecule, (*b*) by improvement of the dosing schedule, or (*c*) by additional treatment with substances which might reduce the adverse effects, then useful results of definite clinical value might be obtained.

Treatment with chloroethylamines makes it possible to produce effects in tumours which are disseminated or localized in deep-seated tissues where radiation therapy is difficult. There have been indications that cases of Hodgkin's disease which have become resistant to X rays have responded to treatment with chloroethylamines.

### ANIMAL EXPERIMENTS.

The animal experiments have indicated the importance of adequate dosage with substances of this type. If C3H mice with spontaneous mammary tumours or grafted lymphosarcomata, or rats with the Walker carcinoma, are given daily doses of 0·1 mg. per kg. of methyl-*bis*(β-chloroethyl)amine hydrochloride (HN2) by subcutaneous or intraperitoneal injection, negligible inhibition of tumour growth is obtained. With higher doses of the order of 1 mg. per kg. complete inhibition of the growth of the Walker rat carcinoma was obtained, and inhibition of spontaneous mammary tumours in mice occurred when doses of 0·4 mg. per kg. were given. Some experiments shown in Fig. 1 and 2 illustrate the increased effect of single massive doses over divided doses.

FIG. 1.—Survival times in days of C3H mice after grafting of the lymphosarcoma LSC. The first group of mice received no treatment, while the other groups were injected with methyl-*bis*-(β-chloroethyl)amine, HN2 with different schedules of dosing.

When the mice were given daily doses of *tris*(β-chloroethyl)amine hydrochloride (HN3) subcutaneously the treatment had very little effect on tumour

FIG. 2.—The growth of spontaneous mammary tumours in mice given intraperitoneal doses of *tris*(β-chloroethyl)amine (HN3). The treatment was most effective when given in single large doses.

growth, but when later the same total amount was given in a single dose, inhibition of tumour growth occurred. Such inhibition was of temporary nature, lasting about one week in the case of the Walker rat carcinoma and up to two weeks in mice bearing spontaneous tumours. The effects of HN2, HN3 and dimethyl-β-chloroethylamine on spontaneous tumours are summarized in Table I. Rats bearing the Walker carcinoma have been given weekly subcutaneous injections of HN2—starting with 1 mg. per kg. for the first three weeks, and then reducing the dose to 0·5 mg./kg. at weekly intervals. A proportion (4/10) of rats treated in this way have been kept alive for three months, when all the control animals died within 21 days of grafting the tumour.

A spontaneous lymphosarcoma (L.S.C.) was also used. This tumour on transplantation into C3H mice usually kills the animals within 21 days, not by means of growth of the local tumour, but with extensive invasion of tissues. Treatment with small doses of HN2 had very little effect on survival of the mice. If treatment with weekly injections of 1 mg. per kg. HN2 was given commencing 10 days after implantation, the mice lived for from 18 to 35 days (Fig. 1).

Mice with spontaneous mammary tumours and mice with the grafted lymphosarcoma (L.S.C.) were treated with doses of 100 mg./kg. of dimethyl-β-chloroethylamine hydrochloride by subcutaneous injection. Such treatment when given repeatedly caused inhibition of some spontaneous mammary tumours (Table I), but had very little effect in prolonging the life of mice with transplanted lymphosarcoma. This compound given in single doses of 100 mg. per kg. to mice caused continuous tremor and incoordinated movement for several days.

Neurotoxic effects were never seen with mice dosed with HN2 or HN3 in doses up to 1 mg. per kg., although larger doses of both substances which have been hydrolysed in neutral solution will cause rapid death with convulsions (Boyland, 1946).

An interesting effect noticed in mice was the bleaching of hair at the site of injection (Fig. 3). It seems that subcutaneous injection of HN2 inhibits pigment formation in the skin of black or brown mice at the site of injection without having any effect on the growth of hair. The effect is similar to that induced by radioactive substances ; it is identical with the phenomenon of depigmentation in mice caused by subcutaneous implantation of plutonium (Brues, 1947, personal communication). Thus " bleaching of hair-colour " is another example of the radiomimetic actions of the *bis*-chloroethylamines.

The metabolism of Walker carcinoma tissue from rats dosed with 1 mg. per kg. HN2 was studied using the Warburg technique. A reduced glycolysis was found for six days following a single treatment (Fig. 7), with no change in the tumour respiration. The nature of the inhibition of glycolysis has not been examined further, but it is probably due to inhibition of hexokinase or other phosphate-transferring enzymes concerned in glycolysis. Dixon and Needham (1946) found that vesicants—either mustard gas or chloroethylamines—inhibited hexokinase when present in low concentrations, and suggested that vesication was due to this inhibition. The inhibition of mitosis is possibly connected with poisoning of enzymes of the same type either concerned in general metabolism, or possibly in the changes in the nucleic acids or enzymes concerned in phosphate transfer in nucleic acids of the chromosomes.

### CYTOLOGICAL EFFECTS.

The cytological effects induced by chloroethylamines were studied in transplanted Walker rat carcinoma. The animals were injected intraperitoneally with 1 mg. per kg. body weight 4–10 days after tumour implantation, and killed at intervals of from 6 hours to 5 days after treatment.

The earliest abnormalities were seen 8 hours after injection. These included the formation of dumb-bell-shaped nuclei (Fig. 5), due to the fact that at telophase the daughter chromosomes could not separate and move to the opposite poles freely, owing to apparent stickiness of the chromosome ends (Fig. 4). Eighteen hours after injection chromosomes in dividing cells fragmented and mitosis remained incomplete (Fig. 6). This effect was most pronounced at 24 hours to 48 hours after treatment, when all dividing cells were abnormal and cell proliferation might be stopped completely. This inhibition lasted for 3–7 days, after which time normal cell division was resumed. When the dose was divided into two equal fractions and injected at 24-hour intervals, the cytological abnormalities were of the same kind as described above, but the damage in individual cells was less severe. Loss of chromosome segments from the nucleus leads to the death of the cell, and is also responsible for the arrest of tumour growth. Treatment of rats with Walker carcinoma with doses of 100 mg./kg. of dimethyl-β-chloroethylamine produced no abnormalities in the dividing cells of the tumour tissue examined 24 and 48 hours after treatment.

The biological effects of nitrogen mustards induced in experimental animals provide a rationale for employing them as chemotherapeutic agents in the treatment of tumours.

FIG. 7.—The glycolysis of Walker rat carcinoma tissue at different times following treatment of the rats with 1 mg. per kg. methyl-*bis*(β-chloroethyl)amine (HN2).

EFFECTS ON BRONCHOGENIC CARCINOMA.

During the past three and a half years some 60 cases of bronchogenic carcinoma unsuitable for other forms of therapy have been treated with various compounds. From 1944 to 1946, 22 patients received either urethane, stilboestrol, dienoestrol, dimethylaminostilbene or 4:4′-diaminodiphenylether. Symptomatic improvement was observed in 1 of the 15 patients receiving urethane, but none of the other drugs had any beneficial effect.

Rhoads (1946) reported a favourable but temporary effect in a case of anaplastic bronchogenic carcinoma receiving methyl-*bis*-(β-chloroethyl)amine, and suggested that further clinical trials were justified. During the first 10 months of 1947, 41 histologically proven cases of bronchogenic carcinoma have been given this compound. Of this group 15 have been referred from the Brompton Neoplastic Clinic, nine from the Royal Cancer Hospital (Free), and the remainder from physicians in other London hospitals.

---

EXPLANATION OF PLATE.

FIG. 3.—Black mouse following three subcutaneous injections each of 0·004 mg. of methyl-*bis*(β-chloroethyl)amine (HN2), showing bleaching or " greying " of hair. Photograph taken six months after the last injection.

FIG. 4.—Cell of Walker rat carcinoma in mitosis 12 hours after injection of HN2. The nucleic acid charge of the chromosomes is altered, causing stickiness of chromosomes. × 1650.

FIG. 5.—Two cells of Walker rat carcinoma with dumb-bell-shaped nuclei, 24 hours after HN2. The daughter chromosomes did not separate, and the two daughter nuclei remain in association. × 1650.

FIG. 6.—Cell of Walker rat carcinoma in anaphase of mitosis 48 hours after HN2. Several of the chromosomes are fragmented, the fragments are left scattered in the cytoplasm and will be lost from the nuclei. × 1550.

3 4 5 6

Boyland, Clegg, Koller, Rhoden and Warwick.

Unsuitability for surgery implies that the growth is not resectable, and for radiation therapy that the patient is too ill for such treatment, that there is evidence of extension of the disease beyond the mediastinum, or that there is fever or pleural exudation. The prognosis in cases unsuitable for surgery and radiation therapy is indicated by the fact that of an untreated group of 50 clinically similar cases, one-third died within 1 month and none lived beyond 8.

In this group, one-third were dangerously ill, being bed-ridden with the disease. The sex incidence and frequency distribution of histological type were similar to those of a total group of 194 seen at the Brompton Neoplastic Clinic.

Thirty-three patients had no previous treatment. Of the remainder, 3 had exploratory thoracotomy, 1 had pneumonectomy with later appearance of secondaries, and 4 had received radiation therapy which had been either clinically unsuccessful, or had been followed in a few months' time by recurrence of symptoms and progression of the disease.

*Form of treatment.*

All patients in this group except 1 received methyl-*bis*-(β-chloroethyl)amine hydrochloride (HN2). A variant of this compound, dimethyl-β-chloroethylamine hydrochloride, was used in 3 other cases, reported here because of the favourable response in 1 case. The compound HN2 was given intravenously in a dose of 0·2 mg. per kg. on two consecutive days in 30 cases, in 10 of which treatment was repeated once or several times at 3-week or longer intervals depending upon the length of remission from symptoms. Two patients received 1 single injection of 0·2 mg. per kg., and two received 1 injection of 0·4 mg. per kg. Four patients received injections of 0·2 mg. per kg. at weekly or 10-day intervals for periods of up to 10 weeks. In the cases receiving the dimethyl compound, 3 mg. per kg. was given the first day and a dose of 5 mg. per kg. the following day.

Several of the ambulant cases received treatment as out-patients, and it would seem that there is no contraindication to such treatment, provided the patient can be returned to his home within an hour of the injection.

*Adverse effects of treatment.*

No serious reactions have been observed in this group. In almost all patients Table II) nausea and vomiting follow injection one to two hours later and

TABLE II.—*Adverse Effects of HN2.*

| | |
|---|---|
| Total number of injections | 152 |
| Nausea | 130 |
| Vomiting | 121 |
| Anorexia | 94 |
| Weakness | 15 |
| Drowsiness | 13 |
| Light-headedness | 7 |
| Thrombophlebitis | 4 |
| Diarrhoea | 2 |
| Rigor | 2 |

continue in most cases for 3–4 hours. In the majority anorexia persists for 24 hours. Feelings of weakness, drowsiness and lightheadedness may occur. In 1 patient such symptoms persisted for 1 week. Thrombophlebitis at the site of the injection, of no serious consequence, followed 4 of over 150 injections. Diarrhoea was remarked upon by 2 patients, and lasted for 3 and 24 hours respectively. Rigors lasting a few minutes have followed 2 injections.

With a dosage of 0·2 mg. per kg. on consecutive days the effects on the white cell count have not been serious. The majority of patients with advanced bronchogenic carcinoma have white cell counts above 10,000 per c.mm., and such patients do not develop the moderate to severe leucopenia observed in cases of Hodgkin's disease (ApThomas and Cullumbine, 1947). In only 2 of our patients has the white cell count fallen below 4000 per c.mm., in these cases to 1000 and 1800 with restoration to normal counts in 3 weeks' time. The first of these cases had had previous radiation therapy, and at the commencement of treatment had a white blood cell count of 4000 per c.mm. Haemorrhagic manifestations have not been observed in this group, nor has any significant change in haemoglobin been noted. Dosages of 0·2 mg. per kg. at 10-day intervals can be tolerated for periods of at least 10 weeks, as judged by the 4 cases we have treated over such a period. The presence of fever, pleural exudation or jaundice are not, in our experience, contraindications to the use of the drug.

The adverse effects of the dimethyl compound were observed in 3 patients, 2 of whom are not included in this group as their diagnosis was not histologically proven. Each of these patients had flushing at the time of injection. Nausea and vomiting were similar to that following the use of HN2, but neurological effects were seen. Patients stated that for several days they could not taste their food; one was confused and disoriented for 5 days, and another, besides being disoriented, was irrational and had incontinence of urine.

*Effects of treatment.*

Twenty-five of the cases reported in this group are still living and have been under observation from 4 to 10 months. Sixteen patients have died.

In estimating the effects of treatment this group may be divided into four classes:

(1) Showing symptomatic relief with objective signs of improvement (18).
(2) Showing symptomatic relief only (6).
(3) Showing objective signs of improvement with no symptomatic relief (1).
(4) Showing no apparent effect (16).

Remissions when they occur usually last 2 to 12 weeks. Further injections produced similar results in most cases. One patient was carried on for a period of 5 months with marked relief of pain, cough and dyspnoea and decrease of sputum for 18 to 21 days following each course of injections. He then showed no further response to the drug and died soon thereafter. A patient with remission of symptoms for 6 to 8 weeks after each treatment is still being observed after 9 months. One patient receiving dimethyl-β-chloroethylamine is still in remission 7 months after treatment and leading a normal life. Two other patients, however, not histologically proven, and therefore not included in this report, were also given the same drug with no apparent beneficial effect.

It is noteworthy that over half of the patients have had relief from their most distressing symptom. The effect on pain (Table III) is remarkable in some

TABLE III.—*Symptomatic Relief following HN2.*

| | | |
|---|---|---|
| Total number of cases | 41 | |
| Symptomatic relief | 24 | |
| | Number of cases. | Relief obtained. |
| Pain | 23 | 17 |
| Cough | 25 | 16 |
| General malaise | 27 | 16 |
| Anorexia | 23 | 14 |
| Dyspnoea | 22 | 11 |
| Hoarseness | 8 | 0 |

cases, relief being experienced within 8 to 48 hours. The pain referred to as being "deep in the chest" is the type most commonly relieved, in contrast to the more localized pain of pleural origin.

Cough is often decreased in severity and may cease altogether. The relief of dyspnoea in some cases is almost complete—an observation very difficult to explain, for in such cases there may not be corresponding observable changes in the lungs.

Relief of general malaise and anorexia is also noted in over half the cases following the initial 1-or 2-day period of adverse effects. Improvement in hoarseness, secondary to involvement of the recurrent laryngeal nerves, did not occur in any of the cases.

*Objective signs of improvement.*

Approximately one-half of the patients (Table IV) had some measurable beneficial effect of treatment, and in all except one of these some symptomatic relief was also experienced.

TABLE IV.—*Objective Improvement following HN2.*

| | | |
|---|---|---|
| Total number of cases | | 41 |
| Objective signs of improvement | | 19 |
| | Number of cases. | Effects noted. |
| Decrease, caval obstruction | 5 | 3 |
| Decrease, sputum | 30 | 18 |
| Decrease, pleural exudate | 13 | 5 |
| Decrease, metastatic tumour size | 10 | 4 |
| Increase, weight | 22 | 9 |
| Radiological improvement | 35 | 8 |
| Remission, physical signs (chest) | 39 | 9 |
| Remission, neurological signs | 10 | 1 |

Of 5 patients with obstruction of the superior vena cava, swelling of the arms disappeared in 1. In 2 others who were moribund at time of treatment regression of swelling to one-half the original size was noted within a week of treatment.

Sputum usually increases in amount for several days, and is said "to come up more easily." The amount may then fall off rapidly, in some cases from 90–120 ml. to 15 ml. or *nil* within a week. The sputum may also change in character from purulent to colourless.

Reabsorption of pleural exudate or decreased rate of formation has been seen in 5 of 13 cases. These observations are of interest in that most radiotherapists hesitate to give treatment to cases with pleural exudation because of the tendency to increase the amount of exudate.

Superficial metastases of measurable size decreased in 3 of 10 cases. A larger total number for such observations would have been obtained had not metastatic nodules been removed for microscopic study at various intervals following injection in 8 cases.

Twenty-two patients who had weight loss were weighed weekly following treatment. In 9 of these cases weight loss continued for approximately 1 week, following which a gain in weight occurred amounting usually to 2 to 8 lb. Four patients gained 10 to 15 lb. in 2 months, and 1 patient who lost 40 lb. in the 18 months prior to treatment with dimethyl-β-chloroethylamine has in 7 months regained all her lost weight.

Radiological evidence of decrease in size of pulmonary metastases and re-aeration of segments or lobes was observed in 8 of 35 cases. Such changes may happen within 5 to 7 days.

A favourable change in physical signs of the chest was noted in 9 of 39 cases, and included those cases with re-aeration of parts of the lung or decrease in pleural exudate.

As already mentioned, recovery of function from paralysis of the recurrent laryngeal nerves has not been noted. In one patient, however, with involvement of the twelfth cranial nerve, complete recovery of function occurred in 9 days and lasted for 7 weeks. Unfortunately, repetition of treatment was not possible in this case.

The appearance of primary tumours at bronchoscopy several days to several weeks following treatment has in the 6 cases observed been unaltered in 3 compared with previous examination. In 3 other cases the tumour area was said to be clearer and to bleed less readily, although a change in size was not observed.

Histological examination of primary or metastatic growth prior to and at various intervals following treatment was carried out in 8 cases. In all post-treatment specimens a great deal of necrosis was present, but one cannot say that such appearances are the result of therapy, for necrosis was seen in many of the pre-treatment specimens as well. Fragmentation of the chromosomes of dividing cells as described by Koller (1947) was not observed in 2 of the 8 specimens.

*Case reports.*

*Case* 1.—J. H—, male, aged 65. Seen first at neoplastic clinic December 13, 1946.

Symptoms : Cough, sputum, general malaise. Anorexia and dyspnoea on exertion for 4 months. Loss of weight—33 lb. in one year. Poor general condition ; signs of consolidation and atelectasis right lower lobe. Small firm gland in right supraclavicular fossa. Weight 111 lb. Sputum, 90 ml. per day muco-purulent. X ray chest December 13, 1946, showed collapse and consolidation right lower lobe.

Bronchoscopy December 19, 1946. Ulcerated stricture of the right lower bronchus at the level of the middle bronchus almost obliterating the lumen. In view of the patient's age, poor general condition and the presence of small firm gland in the right supraclavicular fossa, operation contraindicated.

Pathological report : Poorly differentiated squamous carcinoma.

The presence of a supraclavicular gland made this patient unsuitable for X ray therapy and he was referred for chemotherapy. Two doses of methyl- *bis*-(chloroethyl)amine (0·2 mg. per kg.) given on two consecutive days were followed by nausea and vomiting after each injection. Immediately after the first injection the patient had a rigor for 5 minutes. Temperature 101°. Forty-eight hours after second injection the cough decreased, dyspnoea was much improved and the sputum decreased to 15 ml. per day. Appetite improved, and following initial loss of weight of 3 lb. he gained 8 lb. in one month. The cough and sputum started to increase again in 8 weeks' time but dyspnoea did not return. Radiological examination revealed no changes in the lungs following therapy. Treatment has been repeated on two occasions since with corresponding remission of symptoms and decrease of sputum. Present weight is 108 lb. and general condition remains about the same as when first seen, there being no clinical or radiological evidence of progression of the disease.

*Case* 2.—L. P—, female, aged 47. Admitted to the Royal Cancer Hospital for treatment by Mr. R. W. Raven, April 24, 1947.

Symptoms : Cough with sputum, pain in the chest and dyspnoea 16 months. Loss of weight—40 lb. in 16 months. Patient moribund and emaciated, lying curled in bed without any interest in surroundings. Signs of collapse of right lung with mediastinal shift. Pulse 100. Respirations 24 per minute. Weight 81 lb. Sputum 30 ml. purulent and foul-smelling. Hb, 55 per cent. R.B.C., 3,800,000. W.B.C., 12,600.

X ray of chest April 8, 1947. Heart, mediastinum and trachea displaced to the right; appearance of atelectasis with probable cavitation near 2nd right rib. Bronchoscopy report from Harefield in April, 1946. Carina broadened and invaded on the right by a large nodular friable mass completely occluding the right main bronchus.

Pathological report : Adenocarcinoma with numerous mitotic figures.

Treatment : Dimethyl-chloroethylamine 2 mg. per kg. first day and 5 mg. per kg. second day. Flushing was noticed immediately after each injection. Nausea and vomiting were slight, and there was a feeling of numbness and "largeness" of the tongue with loss of taste for four days. Within one week pain and cough had disappeared. Dyspnoea at rest disappeared in two weeks. The sputum at first increased in amount and lost its bad taste, then decreased, with the cough finally disappearing. General condition slowly improved and in three months the patient was up. Weight steadily increased until 40 lb. was gained. Haemoglobin rose to 87 per cent. The only symptoms at present are a slight cough with a little colourless sputum and dyspnoea on exertion. The X ray appearances are unchanged, but the patient is leading a normal life and looking after her own home.

### DISCUSSION.

The findings are of some theoretical interest in that a demonstrable clinical effect of the chloroethylamines on bronchogenic carcinoma in man has been established. Other workers have been unable so far to demonstrate a corresponding effect on other types of carcinoma in human patients. This probably indicates some tissue specificity of action. It must be remembered, however, that patients are acutely conscious of symptoms referable to the chest and of their degree of severity. It may be that in bronchogenic carcinoma a slight diminution in size of a tumour or amount of tissue reaction around it is sufficient to afford considerable relief from the distressing symptoms. A corresponding effect in a tumour of the breast might reasonably be expected not to be noticed by the patient. In support of such a suggestion is the fact that a similar relief of respiratory symptoms following chloroethylamine without radiological evidence of changes in the lungs has been observed in a case of carcinoma of the breast with pulmonary metastases. On the other hand, metastatic nodules of bronchogenic carcinoma have been seen to decrease in size or even disappear, whereas such changes have not been observed with other types of carcinoma similarly treated at the Royal Cancer Hospital.

From a therapeutic standpoint it can be stated that little has been added to the satisfactory handling of cases of carcinoma of the lung. Impressions based on this study are that the use of the drug may be of some value in cases unsuitable for other forms of therapy, where relief of distressing symptoms may make the patient's remaining days more tolerable. Such advantages, however, should be weighed against the probable amount of discomfort which will be caused by treatment.

There is no definite evidence that the drug alters the progressive course of the disease in the selected group of patients studied, although in the case histories presented it would seem that treatment had a very favourable result, which would not have been expected to occur by chance alone.

The results of further investigations with different dosages of these drugs and variations in the schedule of administration, of the effects in a group of less advanced cases and in a series at present being treated by combination of the drug with radiation therapy, should establish whether these compounds are of more than temporary palliative value. If a palliative remedy is employed then it should be employed continuously at the optimum level of dosage. This dosage remains to be determined.

### SUMMARY.

Methyl-*bis*(β-chloroethyl)amine hydrochloride (HN2) and *tris*(β-chloroethyl)-amine (HN3) produce many effects similar to those caused by X rays, including the greying of hair of mice at the site of injections.

Injection of HN2 or HN3 in adequate doses into animals with particular tumours causes temporary inhibition of tumour growth. This inhibition is accompanied by a reduction in tissue glycolysis. These substances have nucleotoxic actions, as shown by apparent increased stickiness and fragmentation of chromosomes which lead to the death of the injured cell. By repeating the treatment at suitable intervals the life of animals with tumours can be prolonged, but cessation of treatment leads to recurrence of tumour growth.

Forty-one cases of histologically proven carcinoma of the bronchus unsuitable for other forms of therapy have been treated with the chloroethylamines. No serious adverse reactions have been noted. Symptomatic relief and objective signs of improvement were noted in approximately one-half of the cases.

Remissions, when occurring, have lasted usually from two weeks to three months. The theoretical interest of the apparent specificity of chloroethylamine in bronchogenic carcinoma and the indications for its possible use as a palliative measure in advanced cases of the disease are discussed.

The authors wish to express their thanks to the physicians and surgeons of the Brompton Hospital, the Royal Cancer Hospital (Free), St. Thomas's Hospital, the British Post-Graduate School, St. Mary's Hospital, Guy's Hospital, the London Hospital and Saint Bartholomew's Hospital for their interest in the cases reported. We are also indebted to Dr. Jean Watkinson for her observation of cases treated between 1944 and 1946, and to Mr. P. N. Cowen and Dr. J. G. Carr for supplying a mouse with lymphosarcoma.

The authors wish to acknowledge the generous support of the British Empire Cancer Campaign, the Jane Coffin Childs Memorial Fund and the Anna Fuller Fund.

### REFERENCES.

ApThomas, I., and Cullumbine, N.—(1947) *Lancet*, i, 899.
Boyland, E.—(1941) *Biochem. J.*, **35**, 1283.—(1946) *Brit. J. Pharmacol. Chem.*, **1**, 247.
Dixon, M., and Needham, D. M.—(1946) *Nature*, **158**, 432.
Gilman, A., and Philips, F. S.—(1946) *Science*, **103**, 409.
Goodman, L. S., Wintrobe, M. M., Dameshek, W., Goodman, M. S., Gilman, A., and McLennan, M. T.—(1946) *J. Amer. med. Ass.*, **132**, 126.
Jacobsen, L. O., Spurr, C. C., Burton, B. S. G., Smith, T. R., Lushbaugh, C., and Dick, G. F.—(1946) *Ibid.*, **132**, 263.
Koller, P. C.—(1947) *Brit. J. Radiol. Supplement*, **1**, 84.
Rhoads, C. P.—(1946) *J. Amer. med. Ass.*, **131**, 656.
Wilkinson, J. F., and Fletcher, F.—(1947) *Lancet*, ii, 540.